# Myocardial Biomechanics and the Consequent Differentially Expressed Genes of the Left Atrial Ligation Chick Embryonic Model of Hypoplastic Left Heart Syndrome

**DOI:** 10.1007/s10439-023-03187-0

**Published:** 2023-04-09

**Authors:** S. Samaneh Lashkarinia, Wei Xuan Chan, Efthymios Motakis, Sheldon Ho, Hummaira Banu Siddiqui, Mervenur Coban, Bortecine Sevgin, Kerem Pekkan, Choon Hwai Yap

**Affiliations:** 1 Department of Bioengineering, Imperial College London, South Kensington Campus, London, UK; 2 The Jackson Laboratory for Genomic Medicine, Farmington, CT USA; 3 Department of Biomedical Engineering, National University of Singapore, Singapore, Singapore; 4 Department of Mechanical Engineering, Koc University, Istanbul, Turkey

**Keywords:** Embryonic heart, Biomechanics, Hypoplastic left heart syndrome, Mechanobiology, Single-cell RNA sequencing

## Abstract

Left atrial ligation (LAL) of the chick embryonic heart is a model of the hypoplastic left heart syndrome (HLHS) where a purely mechanical intervention without genetic or pharmacological manipulation is employed to initiate cardiac malformation. It is thus a key model for understanding the biomechanical origins of HLHS. However, its myocardial mechanics and subsequent gene expressions are not well-understood. We performed finite element (FE) modeling and single-cell RNA sequencing to address this. 4D high-frequency ultrasound imaging of chick embryonic hearts at HH25 (ED 4.5) were obtained for both LAL and control. Motion tracking was performed to quantify strains. Image-based FE modeling was conducted, using the direction of the smallest strain eigenvector as the orientations of contractions, the Guccione active tension model and a Fung-type transversely isotropic passive stiffness model that was determined via micro-pipette aspiration. Single-cell RNA sequencing of left ventricle (LV) heart tissues was performed for normal and LAL embryos at HH30 (ED 6.5) and differentially expressed genes (DEG) were identified.After LAL, LV thickness increased by 33%, strains in the myofiber direction increased by 42%, while stresses in the myofiber direction decreased by 50%. These were likely related to the reduction in ventricular preload and underloading of the LV due to LAL. RNA-seq data revealed potentially related DEG in myocytes, including mechano-sensing genes (Cadherins, NOTCH1, etc.), myosin contractility genes (MLCK, MLCP, etc.), calcium signaling genes (PI3K, PMCA, etc.), and genes related to fibrosis and fibroelastosis (TGF-β, BMP, etc.). We elucidated the changes to the myocardial biomechanics brought by LAL and the corresponding changes to myocyte gene expressions. These data may be useful in identifying the mechanobiological pathways of HLHS.

**Supplementary Information:**

The online version contains supplementary material available at 10.1007/s10439-023-03187-0.

## Introduction

Hypoplastic left heart syndrome (HLHS) is a congenital heart malformation characterized by an underdevelopment of the left ventricle (LV) and ascending aorta. Thus, no blood is supplied to the systemic circulation. This is a clinically significant disease as it accounts for 2–3% of congenital heart malformations [15, 32] and has the highest mortality rate among all congenital heart defects [14]. Complex palliative surgical repairs are possible but incurs an enormous medical expenditure with poor survival [6]. Even with impressive advancements in surgical procedures, outcomes remain suboptimal and patients cannot sustain a high quality of life [47]. It is thus important to improve our understanding of HLHS pathogenesis and pathophysiology to improve and reduce disease severity.

The etiology of HLHS is currently unclear. It is known that genetic faults cause a minority of congenital heart malformations [13], and offspring of patients who have septal defects are at higher risk of HLHS [27]. However, non-genetic factors such as abnormal hemodynamic and biomechanical loading are also believed to play a major role in causing HLHS, for example, restrictive foramen ovale [8], or aortic stenosis [24] causes flow abnormalities and are associated with HLHS. In cases of aortic stenosis, balloon valvuloplasty fetal heart intervention has been used to resolve the flow obstruction, and has shown promising results in preventing gestational progression to HLHS [11, 12]. These literatures provide substantiation that biomechanics and mechanobiological pathways may be important to HLHS pathogenesis.

Left atrial ligation (LAL) of the chick embryonic heart at early development is a model of HLHS [44]. The ligation is typically performed at HH21, when flow demand to the embryo is doubled. The left atrium is constricted via a 10-0 suture or an atrial clip in LAL procedure. By stage HH31, the LV is underdeveloped while the right ventricle (RV) is enlarged in compensation. At stage HH38, the LV develops fibrosis, bearing similarities to the endocardial fibroelastosis in HLHS patients [25]. LAL relies on mechanical intervention without genetic or pharmacological manipulations. It is therefore a good model for understanding potential biomechanical origin of HLHS.

Hemodynamics of the chick embryonic heart following LAL has previous been investigated by Salman et al. [35], who found that LAL immediately decreased wall shear stresses (WSS) on the atrioventricular (AV) canal, and after septation, through a redistribution of flow to the RV, it caused a further decrease left AV canal WSS and an increase to the right AV canal WSS. We have previously performed image-based flow simulations of the LAL ventricle, and observed that the LAL left ventricle experiences low and oscillatory WSS, which potentially contributes to the hypoplasia [18]. An excellent review on computational investigations of embryonic heart biomechanics with regards to cardiac development is provided by Brown et al. [2].

However, on top of flow biomechanics, the resulting malformation is also likely to be associated with myocardial biomechanics due to LAL, which needs further studies. Tobita et al. [46] measured biaxial passive ventricular stress–strain relations in chick embryos at HH 21 and HH27 after LAL and showed that LAL ventricle is stiffer than the healthy controls. However, the embryonic heart has a complex 3D structure, and there is a need for more detailed and accurate 3D assessment of LAL myocardial biomechanics, as focused in this manuscript.

A previous study have also attempted to characterize the gene expressions in the LV after LAL through microarray analysis, to assist in pinpointing the mechanobiological pathways that may be causing LV hypoplasia [22]. A total of 91 differentially express genes in the LV and 66 gene in the right ventricle were reported. However, the reported gene expressions were not specific to the individual cell types, which requires single-cell RNA seq to achieve, and which may be important for understanding the mechanobiology of the animal model.

In the present study, we analyzed the biomechanics of the normal and LAL myocardium via analysis of myocardial strain function derived from the 2D stacks of high-frequency ultrasound images of embryonic hearts and cardiac finite element (FE) modeling. Further, we performed single-cell RNA sequencing to obtain gene expressions that are potentially important to the mechanobiology leading to the malformation. Our study aims to improve the understanding of biomechanical origins of HLHS, to identify potentially new pharmacological gene targets to treat HLHS, and to inform fetal heart interventions on how to rescue abnormal biomechanics.

## Methods

### Ultrasound Imaging Over 3D Space and Time and Ventricle Wall Assessment

Fertilized White Leghorn chicken eggs were used to obtain embryonic heart images at HH25 (embryonic day 4.5). Animal data were obtained from our previous studies [18, 19], which were approved by the Institutional Animal Use and Care Committee of the National University of Singapore.

The experimental procedures for performing LAL and imaging chick embryonic hearts were provided in our previous publications [18, 19]. Data for 4 normal and 4 LAL embryos were used. Procedures of in ovo LAL are briefly described as follows. At HH21 (embryonic day 3.5), after removing the shell and shell membrane to expose the embryo, the embryo was flipped to the left-side up position, a 10–0 polyamide suture was placed over the left atrium and tightened, and then the embryo was flipped back to the right-side up position and the shell opening was sealed with paraffin film.

**Fig. 1:**
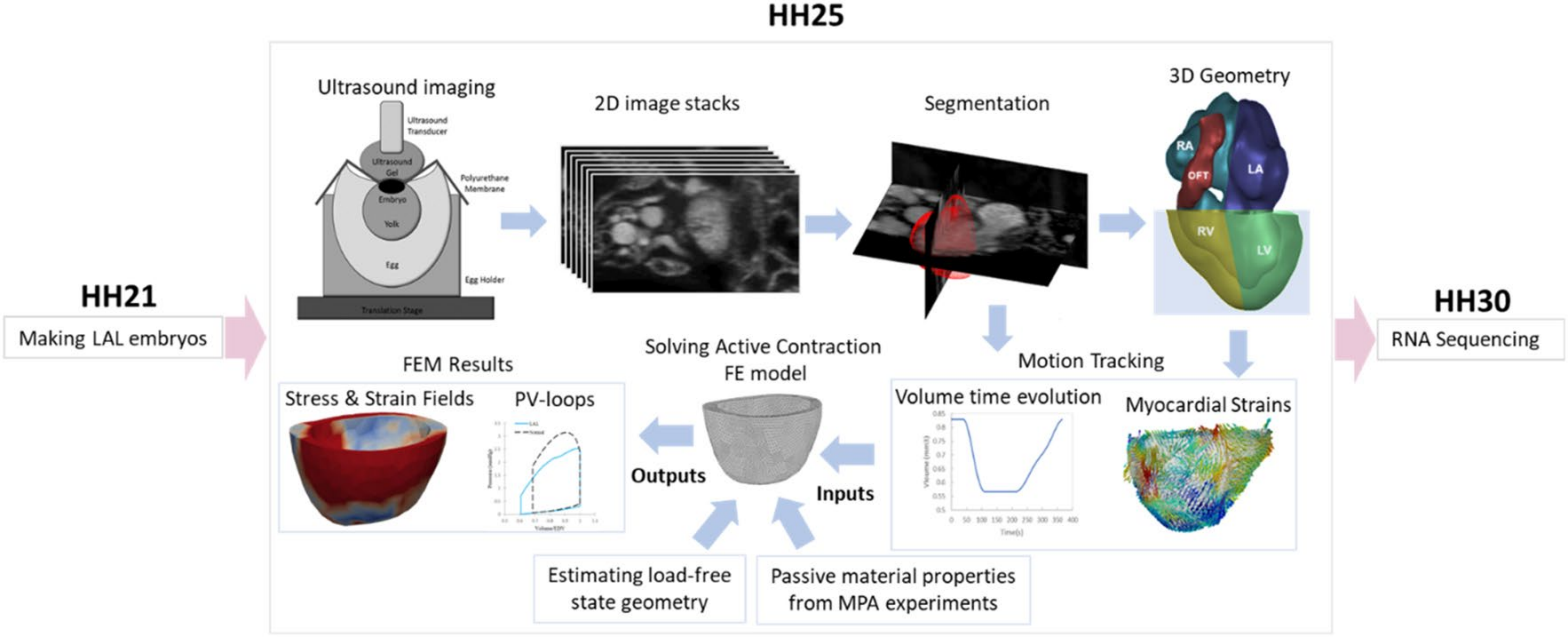
Schematic summary of our methodology. The LAL surgery was conducted at HH21. At HH25, when biomechanical changes manifested, we imaged the embryonic heart, performed segmentation the myocardium, tracked myocardial strains, and performed image-based FE modeling of the heart. At HH30, we performed single-cell RNA sequencing of embryonic LV

Imaging was performed via a high-frequency ultrasound (Vevo2100, Visual Sonics Inc., Canada) with the MS700 transducer (30–70 MHz), with the experimental setup shown in Fig. 1a. A sterilized polyurethane membrane was placed over the exposed embryo. Warmed Aquasonic^®^ 100 ultrasound transducer gel (Parker Corporation, Inc.) was placed over the polyurethane membrane without contacting the embryo. The egg was held by a holder secured onto a micro-adjustable stage, which was used to manually shift the position of the embryo at accurate translational distances. A 75 W infrared heat lamp was placed close to the egg to maintain body temperature during scanning. B-mode images were acquired in a plane-by-plane manner with a regular spacing of 50 microns to cover the 3D space at and around the heart. At each plane, more than 20 cardiac cycles were imaged. This thus formed a stack of 2D cine-images covering a 3D space.

From the images, whole image auto-correlation was used to synchronize timing of different frames to obtain the cardiac phase, while whole image cross-correlation was used to synchronize the timing of neighboring image planes as previously described [17, 19]. No further image registration was required. The quadratic ensemble average (variance of pixels across time) was then calculated to obtain a single cycle to dim tissue spaces and brighten blood spaces, for the segmentation of blood space boundaries or endocardial boundaries. The arithmetic ensemble average (mean of pixels across time) was calculated to get the average cycle that enhances tissue signals for the segmentation of the pericardial boundaries. A custom written lazy-snapping algorithm [42, 49] was used for this segmentation in a slice-by-slice approach, followed by surface reconstruction via VMTK and smoothing via Geomagic Studio^®^. Resulting segmentations are in Supplementary Fig. 1. From the segmentation, an estimate of the average wall thickness of the left and right side of the common ventricle was obtained via manual measurements at 30 random uniformly distributed locations per chamber.

**Fig. 2:**
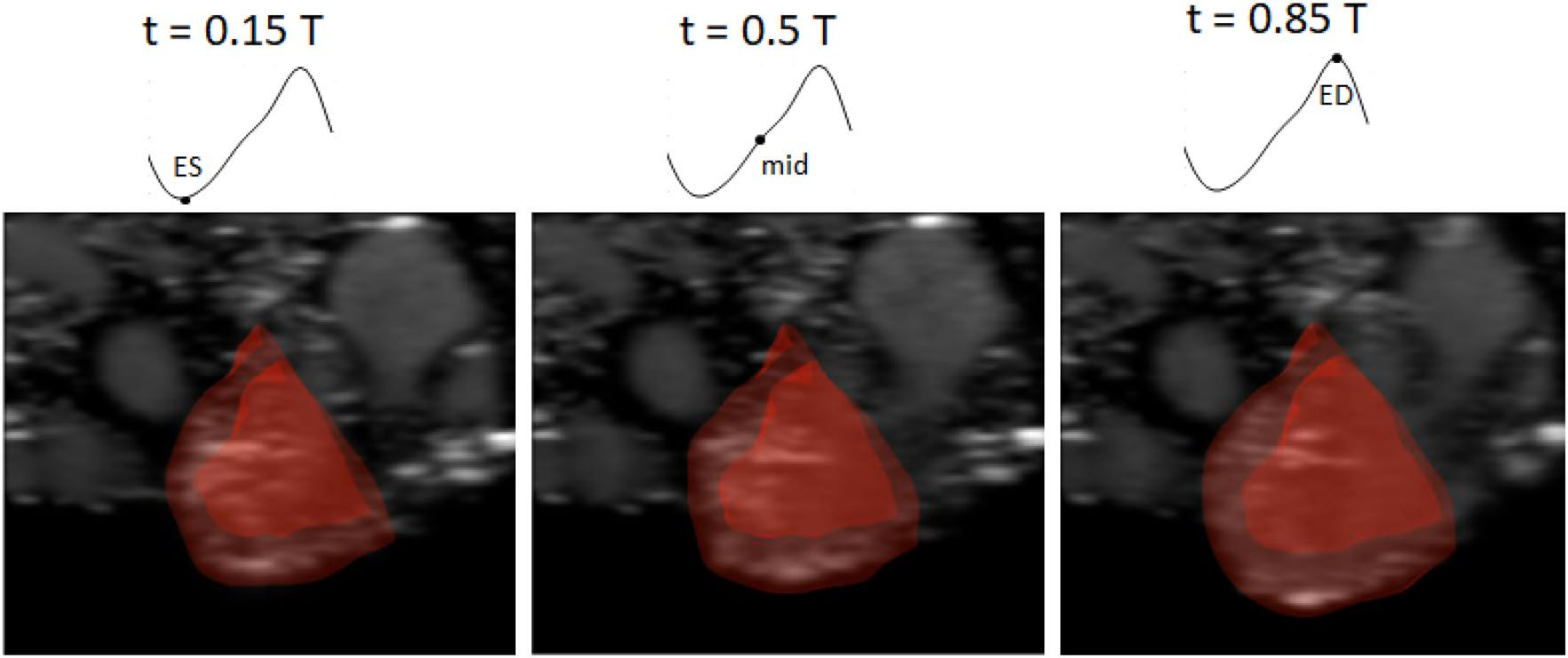
Three-dimensional reconstruction of the chick embryonic ventricle, segmented from 2D stack of ultrasound cine-images for a LAL embryo at HH25. 3D geometries at other time points of the cardiac cycle were re-animated using motion-tracking data. *t* time, *T* cardiac cycle duration, *ES* end-systole, *mid* mid-systole-diastole, *ED* end-diastole

A validated cardiac motion estimation algorithm was used to extract cardiac motions from the arithmetic ensemble averaged echo images [48]. which is available at https://github.com/WeiXuanChan/motionSegmentation. Validation was performed on human adult echocardiography images with MRI truths, chick embryonic heart ultrasound images and zebrafish embryonic heart microscopy images. Figure 2 and Supplementary Video 1 display that this algorithm could successfully capture the motion. In this method, displacement fields are obtained from pair-wise image registration, and a global motion model of Fourier over time and b-splines over space was curve-fitted to the displacement fields, thus achieving cyclic motion regularization and spatial consistency. The algorithm was used to obtain 3D strain information along a layer in the ventricular wall that was 35 μm offset from the epicardial surface. The strain tensor was projected onto the layer surface, and then the principal directions of strain and the principal strain values was quantified, assuming end-diastole as the zero-strain time point. The minimum principal strain eigenvector, projected onto the circumferential-longitudinal plane, represented the direction along which the maximum contraction occurred within the wall plane, and was assumed to be the direction of active tension generation, and was used in the FE model. This was to ensure that the FE cardiac motions matched imaged cardiac motions. We noted that previous publications had used the same approach as indications of myofiber orientations [29, 31].

### Passive Material Properties Measurements

Direct measurements of the stiffness of normal chick embryonic ventricular tissues via micro-pipette aspirations (MPA) was conducted to support realistic FEM simulations, using previously established methods [3]. Fertilized White Leghorn chicken eggs (*Gallus gallus domesticus*) were incubated at 37.5 °C and 65% relative humidity until HH 25 (4.5 days), when the embryonic heart was extracted and washed several times with PBS. The heart tissue was immobilized into the petri dish using syringe needle and it was placed under an optical coherence tomography (OCT) (Thorlabs, Newton, NJ, USA) set-up. A glass capillary pipette (inner radius of 60 μm) controlled by a micromanipulator was brought to the epicardial surface of the ventricle, and vacuum pressure was incrementally applied via a calibrated pipettor (pressure range of 0 to − 60 mmHg). Vacuum pressures were recorded with a servo-null pressure system (WPI, Sarasota, FL, USA) in synchrony with OCT images acquisition, and OCT images were analyzed with ImageJ [36] to measure aspirated tissue length in the pipette. We performed the aspiration experiments on 6 control and 6 LAL samples.

A transversely isotropic Fung-type strain energy from previous cardiac FEM was adopted [28], which is described as:

1 $$W=C\left({e}^{Q}-1\right)$$

$$Q={b}_{ff}{E}_{ff}^{2}+{b}_{xx}\left({E}_{ss}^{2}+{E}_{nn}^{2}+{E}_{sn}^{2}+{E}_{ns}^{2}\right)+{b}_{fx}\left({E}_{fn}^{2}+{E}_{nf}^{2}+{E}_{fs}^{2}+{E}_{sf}^{2}\right)$$

where *E* was the Green–Lagrange strain tensor, and indexes; *f*, *s*, and *n* denoted orthogonal directions corresponding to the myocardial fiber, sheet and sheet normal directions, respectively. This passive mechanical model was scaled to match the MPA data, by altering parameter *C*, but keeping parameters ${b}_{ff}$, ${b}_{xx}$ and ${b}_{fx}$ at our previously published values [28]. To reach the match, we used an iterative inverse FEM approach using Febio software suite (www.febio.org), where the aspiration process was iteratively modeled using experimentally measured vacuum pressures, to find a C value that would provide the same aspirated tissue displacement length within the micro-pipette as observed experimentally. Mesh independence of this FEM was tested by means of the convergence of Cauchy stress ${(\sigma }_{yy})$ to achieve less than 5% change in the results.

### Active Contraction Model in Cardiac Muscle

The active stress generated due to the cardiac muscle contraction is modeled using the previously published Guccione model [16], where active stress in the myofiber direction (${\sigma }_{\text{active}}$) was calculated as,

2 $${\sigma }_{\text{active}}= {T}_{\text{max}}\frac{{\text{Ca}}_{0}^{2}}{({{E\text{Ca}}_{50}^{2}+\text{Ca}}_{0}^{2})} {C}_{\rm t} {e}_{\rm f}\times {e}_{{\rm f}_{0}},$$

where *T*_max_ was the isometric tension in longest sarcomere length. Ca_0_ denoted the peak intracellular calcium concentration. ${e}_{\rm f}$ and ${e}_{{\rm f}_{0}}$ locally defined the active tension generation direction in the current and reference configurations, respectively. ${E\text{Ca}}_{50}$ was the calcium sensitivity variable that depended on myofiber length:

3 $${E\text{Ca}}_{50}=\frac{{\left({\text{Ca}}_{0}\right)}_{\text{max}}}{\sqrt{{e}^{\left(B\left(l-{l}_{0}\right)\right)}-1}},$$

where *B* was a constant that governs shape of peak isometric tension-sarcomere length relation, ${\left({\text{Ca}}_{0}\right)}_{\text{max}}$ was the maximum peak intracellular calcium concentration, and ${l}_{0}$ was the initial sarcomere length at which no active tension develops. $l$ was sarcomere length, which was be calculated as:

4 $$l=\left(\sqrt{{e}_{{\text{f}}_{0}}\cdot {C}_{\text{V}}{e}_{{\rm f}_{0}}}\right){l}_{\text{r}}, \quad {C}_{\text{V}} = {F}_{\text{V}}^{\text{T}} {F}_{\text{V}},$$

where *F*_V_ was the deformation gradient and *C*_V_ was the right Cauchy Green deformation tensor. ${l}_{r}$ was the relaxed sarcomere length. *C*_t_ in Eq. (3), was defined as follows, as a function of time:

5 $${C}_{\text{t}}=\frac{1}{2}\left(1-\text{cos}\omega \right), \omega =\left\{\begin{array}{c}\pi \frac{t}{{t}_{0}}, 0\le t<{t}_{0};\\ \pi \frac{t-{t}_{0}+{t}_{\text{r}}}{{t}_{r}}, {t}_{0}\le t<{t}_{0}+{t}_{\text{r}};\\ 0, {t}_{0}+{t}_{\text{r}}\le t\end{array}\right.$$

where *t*_0_ was the time taken to reach peak tension and *t*_r_ was the duration of relaxation that depended linearly on the sarcomere length $l$ by:

6 $${t}_{r}=ml+b$$

where *m* and *b* are constants. The sarcomere length $l$ was calculated from Eq. (5).

### Finite Element Model of the Chick Ventricle

FE modeling was performed using the open-source library FEniCS (https://fenicsproject.org/). Basal section of the ventricle geometries was cropped off and excluded, and the cropped surface was constrained from moving out of the plane in the FE model. Ventricle geometries were meshed using quadratic tetrahedral elements. Mesh sensitivity analysis showed that about 3000 elements was found to be sufficient to reach < 2% change in the stress results. The spatially varying active tension generation orientations, calculated from image strain analysis via eigenvector analysis, were assigned to mesh node locations where they were measured, but was assumed to be constant across the thickness of the myocardial wall. The volume-time curve from image analysis were used as constraints to the FEM, to ensure that the FEM simulations had a good matched with observed cardiac motions. However, due to smoothing function in the motion tracking, it could not capture isovolumic behavior. This was thus manually enforced, by inserting periods of constant volumes into the waveform, according to iso-volumetric duration measurements by Vos et al. [41], and then performing minor scaling to retain the originally observed stroke volume and cardiac cycle duration.

Before commencing FEM, the load-free geometry of the ventricle was back-computed. Physiological end-diastolic and maximum pressure levels at HH 25 for normal and LAL embryos are interpolated from previous measurements [46]. These end-diastolic pressures, tissue stiffness information from micro-pipette experiments, and end-diastolic volume information from our images were used to compute the load-free geometry. This was done by iteratively simulating diastolic loading from a starting geometry (an initial guess that is close to the load-free geometry) to end-diastole, to back-computed the ventricular pressure at the starting geometry (${P}_{\text{starting}}$) that would allow the simulations to achieve a good match with the targeted end-diastolic pressures and volume. Subsequently, the deformation that would be caused by loading the stating geometry with ${\text{P}}_{\text{starting}}$ calculated, and the inverse of this deformation was applied to the starting geometry to get the estimated load-free stated.

In the model, the initial (*l*_0_) and (*l*_r_) relaxed sarcomere lengths are extrapolated from a published study where sarcomere lengths at HH23 were measured [5]. Heart rate was 164 bpm [41]. Physiological end-diastolic and maximum pressure levels at HH 25 for normal and LAL embryos are interpolated from previous measurements [46]. This was used to determine active tension magnitude, where *T*_max_ in Eq. (2) was adjusted until the peak ventricular pressure from FEM matched measurements from the literature. Minor adjustments were made to *m* and *b* parameters in Eq. (6) to enable a good overall end systolic and early diastolic shape of the pressure–volume loop and to match the end-diastolic pressure levels (values used are given in Supplementary Table 1). All FEM parameters used are shown in Table 1.

**Table 1:** Passive material properties and active contraction ventricle model parameters

Parameter	Unit	Value	
Anisotropic Fung-type material			
*C*	Pa	Normal	19.7
		LAL	27.6
*b*_*ff*_	–	29.9	
*b*_*xx*_	–	13.3	
*b*_*fx*_	–	26.6	
Active contraction ventricle model			
Ca_0_	μm	4.35	
(Ca_0_)_max_	μm	4.35	
*t*_0_	ms	110	
*B*	μm^−1^	4.75	
*l*_0_	μm	1.7	
*l*_r_	μm	2.2	

### Statistical Analysis

Statistical testing was done by first confirming that the data distribution was normal via the Shapiro–Wilk test. Results were expressed as mean ± SD. Wilcoxon non-parametric test was used for hypothesis testing with *p* < 0.05 considered significant.

### Single-Cell RNA Sequencing of Chick Embryonic Heart Ventricles

Single-cell RNA sequencing of chick embryonic heart ventricles for normal and LAL embryos at HH30 (6.5 days) was performed at the Genome Institute of Singapore. This embryonic stage is chosen as it is the known timing of the manifestation of the hypoplastic LV morphology. Tissue dissociation and sorting into single cells were done within 1 h of harvest, and cells were then frozen until all samples were collected, before sequencing is performed. Left ventricle tissue were extracted from embryonic heart and dissociated with Papain Dissociation System Kit (Worthington, Lakewood, New Jersey, USA). The left ventricle tissue was immersed in 500 μl of Papain-DNAse I solution (from the Papain kit) and incubated on a thermomixer at 30 °C and 600 rpm for 45 min and every 15 min to promote dissociation. The Fluidigm (Fluidigm, San Francisco, California, USA) automated microfluidic C1 system was used to isolate single dissociated cells. DAPI were added to the dissociated cardiac cells and were sorted with Fluidigm microfluidic chip. Wells in the Fluidigm chip were inspected via fluorescence microscopy and visual QC was performed to exclude potential doublets or debris captured in the chip capture sites. Single-cell whole-transcriptomic sequencing was performed with the Illumina Hiseq 2500 system (USA). Barcoding of cDNA of each cell was performed such that we could link sequencing results of each cell to the source of the sample. At the read level, our QC pipeline excluded cells with low sequence quality, < 50,000 reads or < 1000 detected genes. 391 cells out of 460 cells (85%) were kept. The data were normalized by library size using Seurat’s log-normalization model “others” [4] and subsequently batch effect corrected with the “removeBatchEffect” function from the “limma R” package to account for the different days of the experiment [33] and the cyclone-estimated cell cycle phases [37]. The cells were clustered with Affinity Propagation [10] after t-SNE dimensionality reduction [1] using the 2000 variable genes. For each cluster k (*N* = 4), Seurat’s Wilcoxon model returned a list of differentially expressed genes for LAL vs Normal (|log2FC| > 0.4 and FDR ≤ 5%), which were used for GO enrichment analysis with amiGO [http://amigo.geneontology.org/amigo]. Seurat plots for each cluster (*N* = 4) is provided in Supplementary Document 1 at 10.6084/m9.figshare.22227772.v2. Cell type annotation was based on detected differentially expressed markers and informative terms from the GO enrichment analysis (FDR < 5%). Out of 391 cells (roughly 50%–50% normal vs LAL), 130 were endothelial cells, 171 were myocardial cells, 59 were fibroblasts, and the rest were “others”.

## Results

### Ventricle Wall Thickness And Strains in the Minimum Principal Directions

**Fig. 3:**
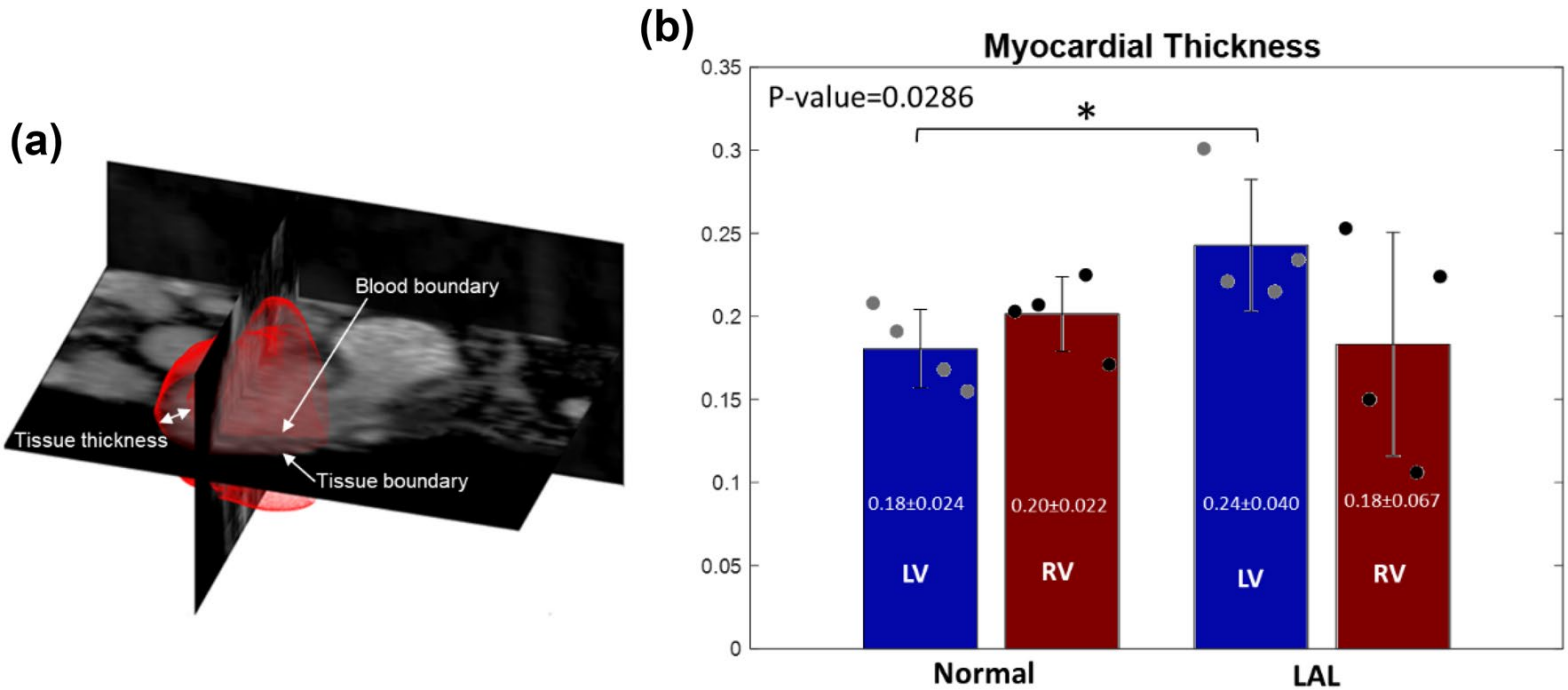
**a** 3D reconstructed ventricle epicardial and endocardial boundary, superimposed onto the quadratic ensemble averaged ultrasound image at HH25. **b** Average ventricle thickness of Normal and LAL hearts for the left and right ventricles at HH25

Figure 3a shows the blood and tissue boundaries in ventricles acquired from ultrasound image analysis in a LAL embryonic heart, while Supplementary Fig. 1 shows that for all samples. As shown in Fig. 3b, mean LV wall thickness was found to be higher for LAL embryos compared to controls (0.24 ± 0.044 mm versus 0.18 ± 0.024 mm, *p* = 0.0286), but mean RV wall thickness did not show statistically significant difference between LAL and controls (0.18 ± 0.067 mm versus 0.20 ± 0.0222 mm, *p* = 0.8857).

**Fig. 4:**
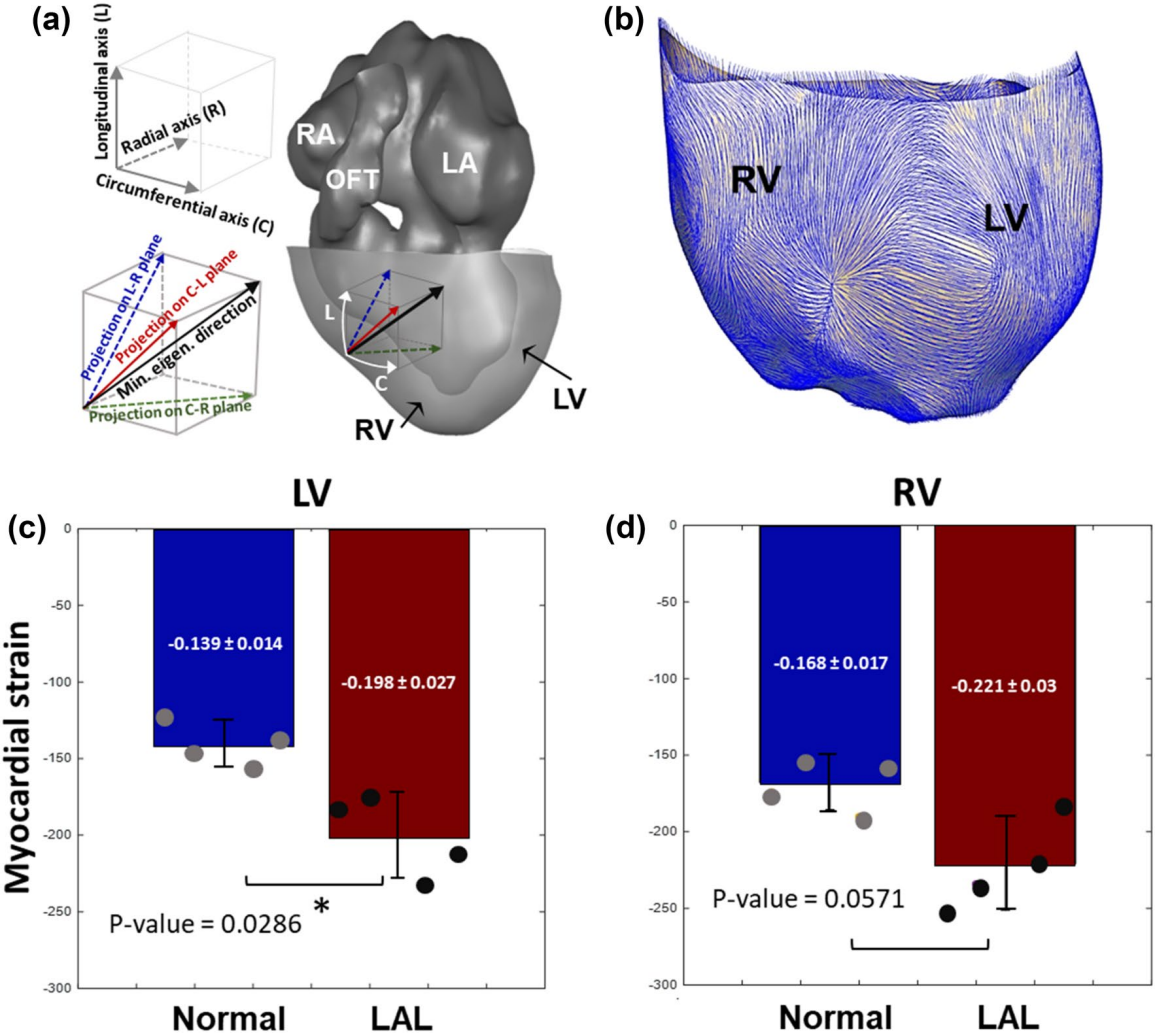
**a** Reconstruction of the blood spaces in the chick embryonic heart at HH25, superimposed on the myocardial segmentation of the ventricle. Definitions of the myocardial circumferential and longitudinal axes are indicated as well. **b** Directions of maximum strain in the ventricle, calculated via the minimum eigenvector of the strain tensor. **c, d** The spatial-averaged strains in the minimum eigenvector direction for normal and LAL hearts in **c** LV and **d** RV respectively. Results were obtained from image motion-tracking

A representative reconstruction of the ventricular myocardium is shown in Fig. 4a, together with schematic definitions of longitudinal and circumferential directions. Image analysis showed that the direction of greatest contractile deformation at HH25 was statistically random, with an almost uniform distribution, and there was no observable difference between normal and LAL ventricles (Supplementary Fig. 2). The directions of greatest contractile deformations are illustrated in Fig. 4b for a representative case. Myocardial strains in these minimum principal directions, after spatial-averaging, are shown in Fig. 4c and d, and were greater in magnitude in LAL embryos compared to the normal embryos for both the LV (− 0.198 ± 0.027 versus − 0.139 ± 0.0114 versus, *p* = 0.0286) and the RV (− 0.221 ± 0.03 versus − 0.168 ± 0.017, *p* = 0.0571), suggesting that contractile deformations were greater in LAL LV than normal LV.

### Myocardial Stiffness

From the MPA measurements, the best matching C parameter in the Fung strain energy function (Eq. 2) was found to be 19.7 ± 10.3 Pa for control and 29.3 ± 7.8 Pa for LAL at HH25 (Table 1). These passive material properties were used in the FEM simulations. Our measurements are in accordance with findings of an earlier study by Tobita et al. [46], in which biaxial passive inflation tests showed that myocardial stiffness constant of exponential material model in LAL embryos were about 40% higher than the normal ones at HH27.

### Peak Active Tension And stress Magnitude in Minimum Principal Directions

**Fig. 5:**
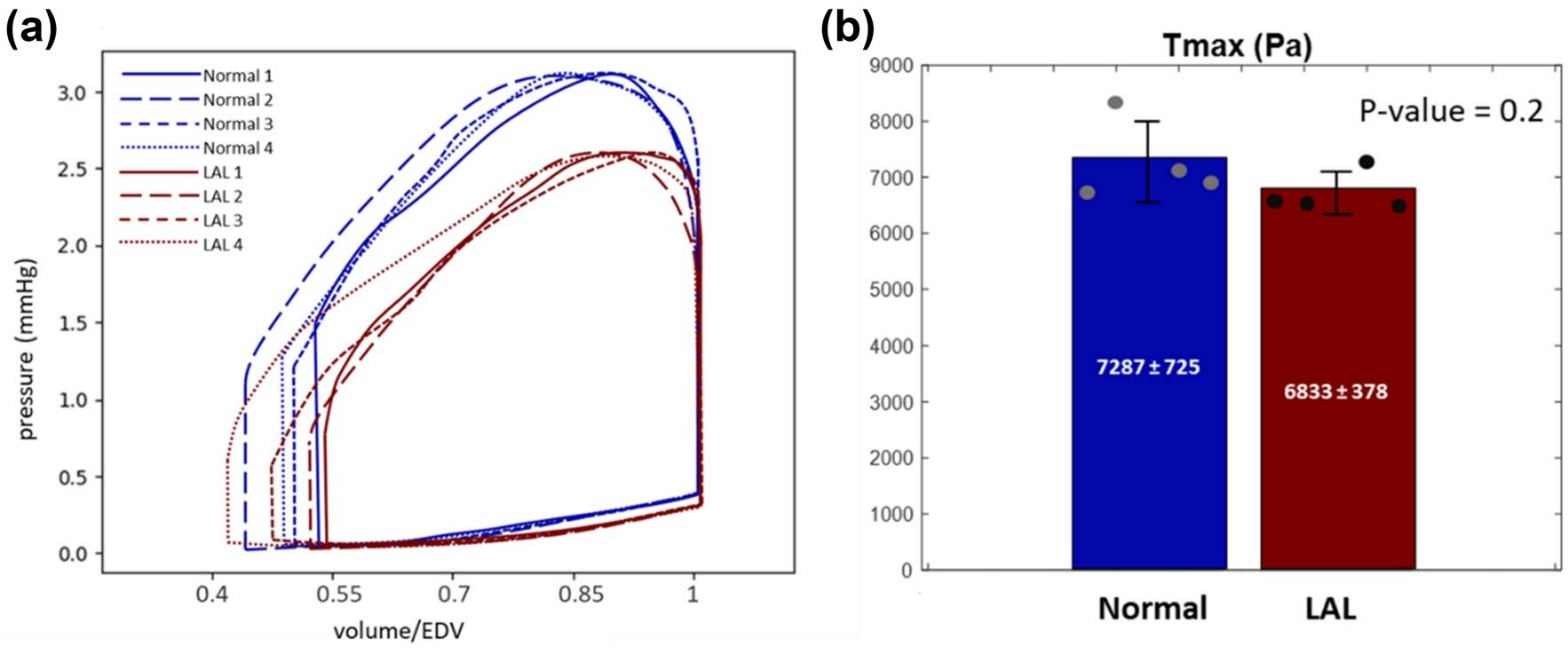
**a** Pressure–volume loops obtained from FE model for 4 normal and 4 LAL samples. Active tension magnitude (${\mathbf{T}}_{\max}$) is tuned for each simulation to satisfy the maximum pressure criteria of ${\mathbf{P}}_{\max}=$ 3.1 mmHg for Normal and ${\mathbf{P}}_{\max}$ = 2.6 mmHg for LAL cases. **b** The myocardial active tension that enabled a good fit to the maximum pressure criteria is compared

Pressure–volume loops obtained from FEM are shown in Fig. 5 for 4 LAL and 4 normal embryonic hearts. Myocardial contractility was tuned to enable peak systolic pressures to reach literature values of 3.1 mmHg in normal hearts and 2.6 mmHg in LAL hearts. The active tension magnitude (*T*_max_) required for this was 6833 ± 378 Pa for LAL hearts, which was 10% less than that for healthy hearts, 7287 ± 725 Pa, even though this was not statistically significant (*p* value = 0.114) (Fig. 5b). These might suggest that LAL myocardium was not as engaged as normal myocardium. End-systolic pressures were specified to literature values of 0.38 mmHg in normal hearts and 0.28 mmHg in LAL hearts, and were used together with imaged end-diastolic volumes to calculate the load-free geometry of each heart.

**Fig. 6:**
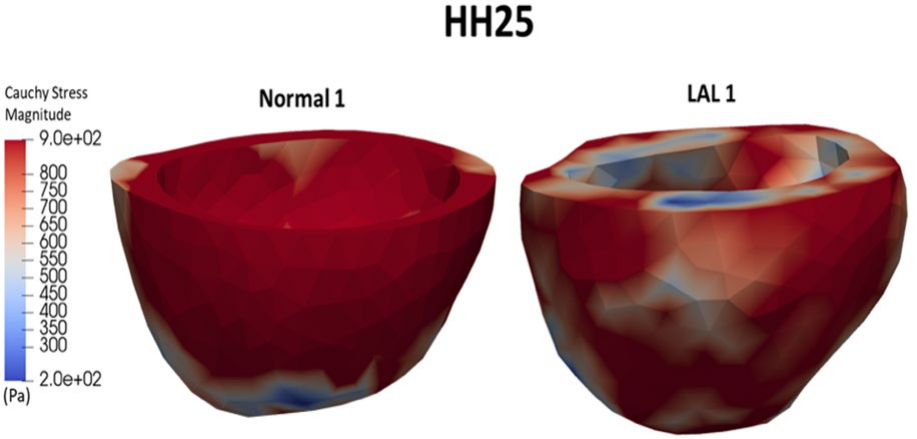
Spatial distribution of Cauchy stress tensor magnitude obtained from FE models, plotted at the end-diastolic time point for samples Normal 1 and LAL 1

**Fig. 7:**
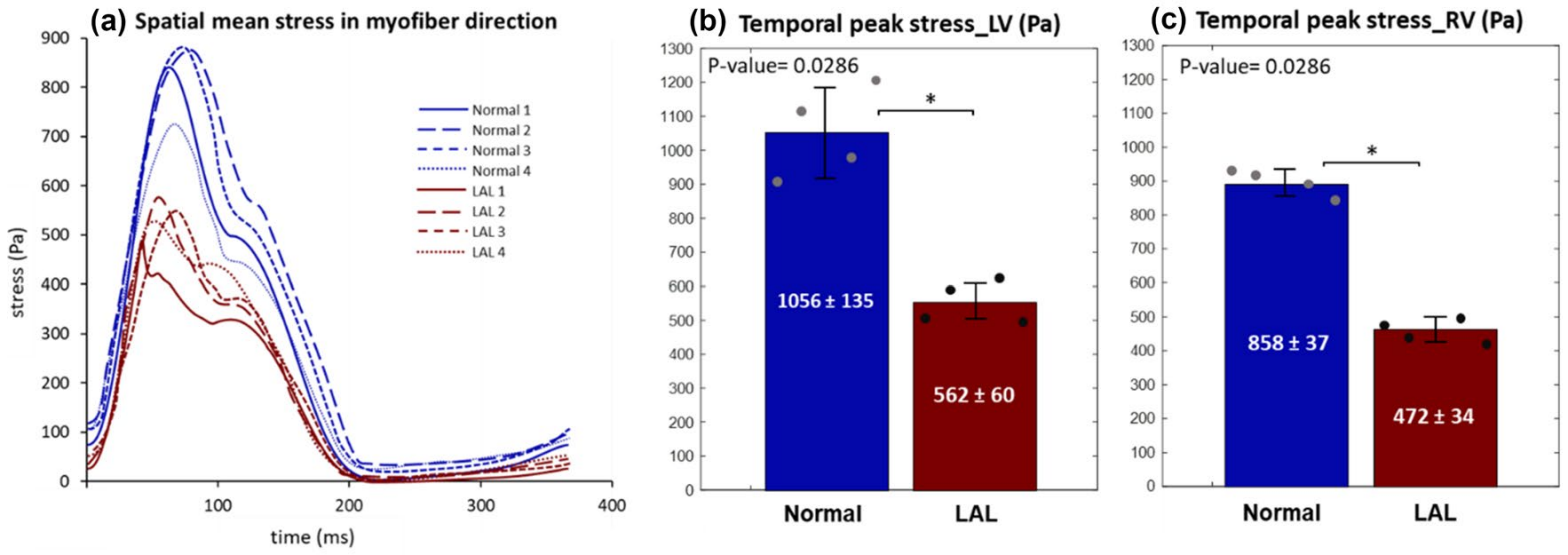
**a** Spatially averaged stress in the minimum eigenvector direction (direction of maximum shortening) obtained from subject-specific FE models, plotted over the cardiac cycle, for 4 normal and 4 LAL chick embryonic ventricles. **b, c** Average temporal peak stress in minimum eigenvector direction in normal and LAL embryos for **b** LV and **c** RV, respectively

Figure 6 shows the spatial distribution of the Frobenius norm of the Cauchy stress tensor (the root of the sum of the square of all components) at the end-diastolic time point for samples Normal 1 and LAL 1, and for the entire cardiac cycle in Supplementary Videos 2 and 3. Results showed the absence of a spatial pattern, and that the Normal heart were under higher stresses than the LAL heart. The spatial mean of stresses in the minimum principal directions (directions of greatest contractile strains) are plotted over the cardiac cycle in Fig. 7. Temporal-peak of myocardial stresses in these directions was higher for normal hearts compared to LAL for both LV (858 ± 37 Pa versus 473 ± 34 Pa, *p* = 0.0286) and RV (1056 ± 135 Pa versus 562 ± 60, *p* = 0.0286), suggesting that LAL reduced the stresses experienced by the myocardium.

### Gene Expression Levels in Normal and LAL

**Fig. 8:**
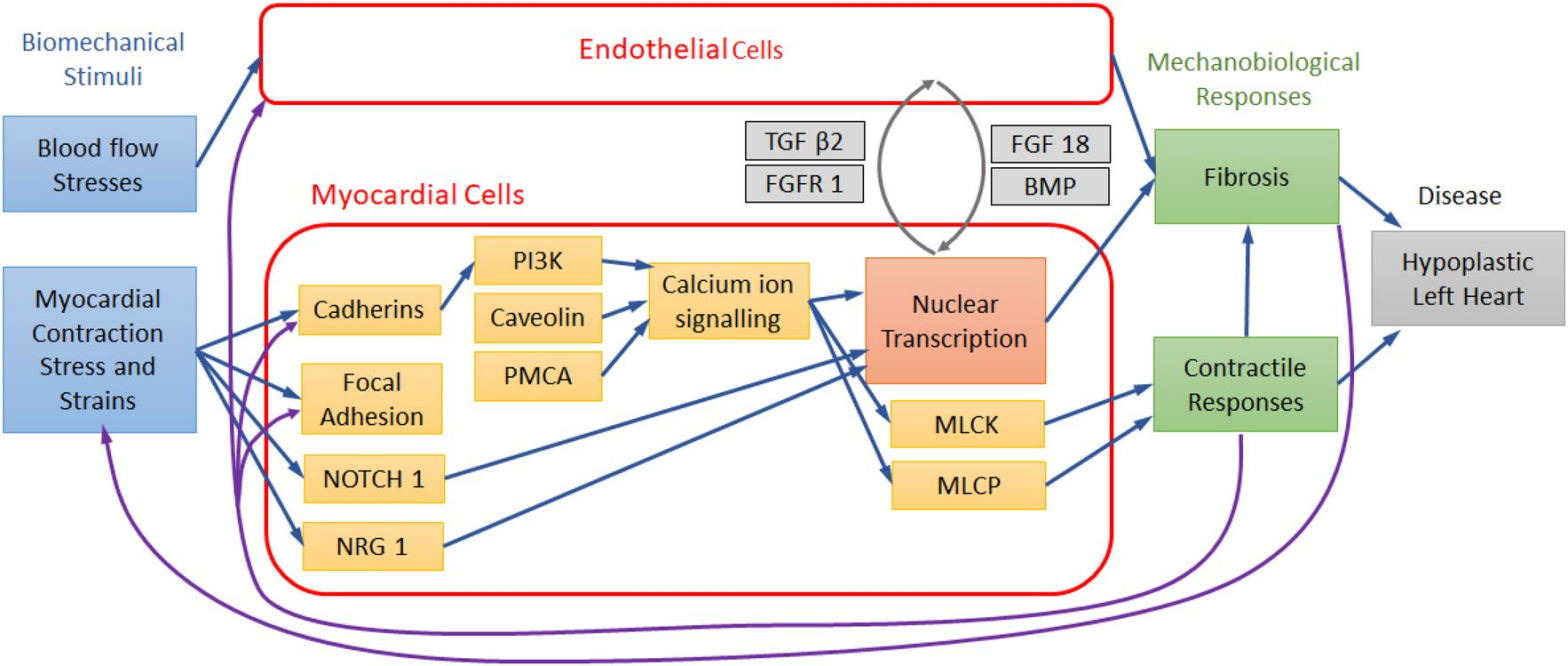
Selected differentially expressed genes obtained from single-cell RNA sequencing of the normal and LAL chick embryonic heart at HH30 that are potentially important for mechanobiological origins of the hypoplastic LV morphogenesis

A list of all differentially expressed genes between normal and LAL LV myocardial cells at HH30 is given in the Table 2, and the raw data including expression levels of all analyzed genes is provided in Supplementary Document 2 (at https://doi.org/10.6084/m9.figshare.22227772.v2). As our current study focus on tissue biomechanics, we analyze only differentially expressed genes for myocytes. However, gene expressions for all cell types are given in Supplementary Document 2. Among the list, we found several differentially expressed genes that were relevant to stress and stress sensing in the myocardium, including focal adhesion genes ITGα and ITGβ, PTK2, SRC and α-actinin, Glypicans, Cadherin and Notch1. Interestingly, NRG1 expression was also altered, collaborating with observed trabeculation compaction changes during LAL [39]. There were downstream expressions related to calcium signaling (PI3K; Caveolin; SERCA; STIM2; PMCA), and some related to myosin contractility (CALM; MLCK, MLCP, PDE). Many ECM remodeling genes were upregulated with LAL (6 types of collagen; laminin, fibronectin, 2 types of LOXL, 3 types of MMP, TIMP3), suggesting myocardial fibrosis or fibroelastosis, which agreed with previous findings in this disease model [30, 40]. We also found evidence of changes to TGF-β (TGF-β2, R3) and BMP (BMP2, 10, R2) expression levels, suggesting changes to their signaling, which may play a role in the fibrosis. Changes to expressions of FGF (FGF 18, R1, R3) and PDGF (PDGF-D, Rα) were also observed, which may play a role in altered proliferation of LV cells. Figure 8 shows a simplified summary of our findings in (Table 2).

**Table 2:** Comparison of gene expressions in normal and LAL at HH30

Gene		Normal	LAL	Log FC	FDR	*p* value	KEGG pathway
Glypican	GPC5	3.36	1.68	− 1.00	1.2e−3	4.9e−4	Mechanosensing
	GPC 2	1.44	3.14	1.13	2.7e−6	6.2e−7	
	GPC 1	80.26	369.73	2.20	7.8e−8	1.3e−8	
Focal adhesion	ITGα1	1.32	3.31	1.33	2.1e−9	2.6e−10	
	ITGα8	1.38	2.28	0.73	3.4e−6	8.1e−7	
	ITGβ3	0.88	1.99	1.18	7.5e−12	4.8e−13	
	ITGβ5	1.18	2.39	1.03	8.7e−8	1.5e−8	
	ITGβ1BP1	6.63	22.90	1.79	1.6e−5	4.2e−6	
	PTK2	1.88	5.76	1.62	1.8e−4	6.2e−5	
	SRC	4.41	13.88	1.65	6.3e−10	6.6e−11	
	ACTN4	1.11	2.04	0.88	9.3e−9	1.3e−9	
Cadherin	CDH5	2.07	7.12	1.78	2.7e−6	6.3e−7	
	CDH11	5.67	18.23	1.68	1.8e−6	4.1e−7	
NOTCH1		39.89	148.79	1.90	6.5e−6	1.6e−6	Mechanosensing/trabeculation compaction
NRG1		14.76	82.12	2.48	5.7e−17	8.8e−19	Trabeculation compaction
PIK3CA		1.79	2.36	0.40	6.4e−5	2.0e−5	Calcium ion signaling
CAV 2		0.97	1.53	0.66	6.2e−13	3.2e−14	
CAVIN 1		45.51	199.82	2.13	1.6e−5	4.2e−6	
CAVIN 2		1.20	1.95	0.70	1.3e−8	1.9e−9	
SERCA/ATP2A3		0.88	1.73	0.98	2.0e−13	9.3e−15	
PMCA	ATP2B1	2.10	4.76	1.18	1.1e−6	2.4e−7	
	ATP2B4	19.15	53.66	1.49	2.8e−4	1.2e−3	
SPCA/ ATP2C1		1.46	1.94	0.41	1.1e−5	2.8e−6	
STIM2		1.71	3.65	1.09	4.6e−7	9.2e−8	
CALM	CALM1	304.46	1002.12	1.72	5.1e−6	1.3e−6	Myosin contractility Proliferation of LV cells
	CALM2	996.45	2721.15	1.45	5.5e−4	1.4e−3	
MLCK		35.71	179.63	2.33	1.2e−7	2.1e−8	
MLCP	PPP1R9A	0.98	1.47	0.59	1.5e−8	2.2e−9	
	PPP1R9B	4.27	13.34	1.65	1.6e−3	6.5e−4	
	PPP1R3C	1.78	5.44	1.61	4.5e−10	4.5e−11	
PDE	PDE10A	1.13	1.75	0.63	2.2e−10	2.1e−11	
	PDE3A	3.70	10.16	1.46	2.8e−4	9.8e−5	
	PDE5A	1.71	4.77	1.48	5.1e−6	1.2e−6	
	PDE4B	16.04	83.94	2.39	6.0e−8	10.0e−9	
FGF18		6.07	21.78	1.84	2.6e−12	1.6e−13	Proliferation of LV cells
FGFR 1		17.03	34.86	1.03	2.0e−3	8.5e−4	
FGFR 3		3.15	11.25	1.83	8.6e−6	2.2e−6	
PDGF-D		3.96	11.51	1.54	2.2e−3	9.3e−4	
PDGF Rα		1.33	3.17	1.26	1.7e−7	3.1e−8	
COL1 A1		21.42	114.67	2.42	3.0e−7	5.7e−8	Fibroelastosis and fibrosis
COL27 A1		15.98	78.15	2.29	3.4e−15	1.1e−16	
COL3 A1		25.75	107.11	2.06	4.0e−7	8.0e−8	
COL6 A2		6.88	28.22	2.04	1.1e−9	1.2e−10	
COL6 A3		2.08	5.75	1.47	2.1e−4	7.1e−5	
COL 12 A1		1.34	3.47	1.37	1.2e−8	1.8e−9	
COL1 A2		29.05	73.56	1.34	4.2e−3	1.9e−3	
COL 6 A1		2.58	4.47	0.79	2.2e−3	9.5e−4	
COL 4 A2		3.69	5.48	0.57	3.7e−3	1.6e−3	
Fibronectin/FN1		315.71	1104.15	1.81	1.4e−7	2.4e−8	
LOXL1		1.45	2.23	0.62	5.9e−3	2.7e−3	
LOXL2		7.72	20.83	1.43	7.3e−3	3.5e−3	
MMP2		2.63	5.54	1.08	1.4e−5	3.9e−6	
MMP16		1.59	3.62	1.19	7.2e−3	3.4e−3	
TIMP3		99.14	380.64	1.94	1.1e−5	2.8e−6	
TGF β 2		47.68	151.12	1.66	5.4e−4	2.0e−4	
TGF β R3		1.20	2.39	0.99	2.5e−3	1.1e−3	
BMP 2		4.01	19.60	2.29	1.5e−9	1.7e−10	
BMP 10		3.37	8.03	1.25	2.1e−3	9.0e−4	
BMP R2		2.06	4.88	1.25	4.4e−5	1.3e−5	

171 myocardial cells were tested and statistical parameters are provided for better interpretation of the results

## Discussion

In the first part of the current study, we performed biomechanical analysis of the chick embryonic ventricle during LAL at HH25, to understand alterations to the myocardial biomechanical loading and deformational characteristics that might be contributing to the hypoplastic LV in the animal model. There has been much interest in inferring myocardial biomechanics of this animal model via imaging, histology, and in vivo manipulations in the past [39, 43, 46]. However, detailed finite element modeling that considers complex 3D anatomy, active tension generation and passive tissue stiffness has not been reported. Our study utilized 4D high-frequency ultrasound images, and image-based FEM simulations to achieve this, to obtain more realistic estimations of biomechanical parameters. In the second part of our study, we collected single-cell RNA seq data of chick embryonic LV at HH30, to understand the gene expression consequent to the biomechanical loading discovered above, and to address a gap in the literature on such data. The raw data results of the sequencing is shared here to inform further investigations of mechanobiological pathways.

Our results indicated that with LAL, LV wall thickness increased while RV wall thickness slightly remain unchanged, myocardial strains were higher, and myocardial stresses and active tension were lower. In terms of thickness, a previous study by Tobita et al. showed that after LAL and at HH27, the compact layer thickness decreased in the RV but remain unchanged in the LV [46], but measurements were based on histology in 2D, and were only on the compact layer, while our current measurements were made in vivo over a 3D space, and included all cardiac wall tissues with sufficient echo signals, which is likely to include some of the trabecular layer on top of the compact layer, making comparisons difficult. A second study by Sedmera et al. has found via histological means that neither the RV nor the LV compact layer thickness changed after LAL at HH29 [39], which did not agree with some of Tobita et al.’s measurements. These collective discrepancies suggested high variability in wall thickness. In terms of strains, our study found that mid-wall myocardial strains were higher in both ventricles after LAL at HH25. This corroborated an earlier study by Tobita et al. [45], which found that at HH27, peak systolic epicardial circumferential strain was significantly higher in LAL LV compared to control LV but there was no difference in the corresponding longitudinal strain.

We propose that our observations can be explained as follows. The reduction of flow from the atria after LAL led to reduced preload and ventricular end-diastolic volume. This led to myofibers being less stretched and thus less engaged in the subsequent contraction phase, in alignment with the Frank-Starling mechanism, thus leading to decreased active tension generation and lower systolic blood pressure. Lower pressures then translated to reduce myocardial stresses. Available data could support this proposal. Our measurements showed that the LAL ventricle was smaller than control ventricle at end-diastole (width of 1.37 ± 0.09 mm versus 1.44 ±0.07 mm) although the difference was not significant, suggesting a mild reduction in ventricular pre-load. Observations in previous studies supported our proposal, Tobita et al. found LAL LV to be significantly smaller in its major axis length with no change to the minor axis length [46], while Sedmera et al. found that LAL LV and RV to have reduced lengths compared to controls [39]. We further propose the observed higher strain in LAL ventricles is due to lower pressures in the LAL ventricle. LAL ventricles have lower pressure to resist contractile motions, and thus have higher strains.

Interestingly, in a previous study investigating the effects of the unloading of mouse hearts via the transplantation of a mouse heart to another’s abdominal aorta, it was observed that consequent to the pressure unloading of the transplanted heart, there were increased fractional shortening, reduced cell sizes, and reduced contractility [34]. In our study, the embryonic hearts were similarly unloaded due to reduced preload, and we had similar observations of higher strains and reduced contractility.

The above-described biomechanical differences caused by LAL were likely to contribute to altered biological expressions and altered growth and remodeling, leading to the HLHS morphology at HH30. Although one would typically expect that increased LV strain would lead to an enlarged ventricle, however in this case, we speculate that effects of reduced pressure and myocardial stresses dominated, and were the main cause of the LV hypoplasia.

Our studies also revealed that the embryonic heart had a statistical uniform distribution in the directions of contraction across the whole ventricle, and that there was not a distinction between LAL and normal ventricle at HH25 in this statistical sense. This corroborated findings of the previous study that utilized confocal imaging to quantify myofiber orientations [43], which showed no distinctive difference in transmural fiber orientation between LAL and controls close to this stage.

In terms of gene expressions, we observed significant upregulation several fibrosis genes, including many types of Collagen, in LAL myocardium. These were in line with past reports of myocardial fibrosis [30] and increased myocardial stiffness [45] in the same animal model. This could be due to hypoxia, as past studies showed that LAL experiences myocardial hypoxia due to abnormal hemodynamics, as well as increased collagen I production in the sub-endocardium coupled with significant thickening of the sub-endocardium [30]. Fibrosis could also be elevated via the TGF-β pathway, which were shown to be important fibrosis mechanisms during adult cardiac remodeling and failure [9, 20], as TGF-β2 was found upregulated in our study. We also observed upregulation in BMP genes, which suggested an active counter regulatory mechanism to upregulation of TGF-β genes [26]. However, the prevailing thought on TGF-β regulated fibrosis is that TGF-β upregulation was consequent to elevated mechanical stresses [50], while the LAL myocardium in our study experienced reduced stresses. We speculate that other biomechanical abnormalities could play a role in activating TGF-β mediated fibrosis, such as the elevated strains in LAL myocardium.

An earlier study by Sedmera et al. [39] on chick embryo showed that the under-loading in the LV after LAL resulted in accelerated trabeculae compaction at HH29. Our gene analysis at HH30 showed upregulation of NOTCH1 and NRG1 in LAL myocardium, which were genes known to be important for trabeculation formation and compaction [7, 23]. However, while previous studies showed downregulation of FGF2 at HH34 after LAL [38], we did not observe any differential expression to FGF2 and even noted an upregulation in FGF18 at HH30, although an upregulation of FGFR1 agreed with the aforementioned study. Our data could thus not confirm the lack of myocardial proliferation after LAL, but this could be due to the difference in the developmental stages investigated (HH34 versus HH30).

Ritter et al. showed that increased expression levels of calcium ion signaling genes could be due to the under-loading of cardiac myocytes [34]. This corroborated with our findings in which underloading of ventricle was associated with upregulated Calcium ion signaling genes like PI3K, Caveolin and PMCA expression levels. Chan et al. showed that an overexpression of MLCK resulted in significantly increased cardiomyocyte contractile amplitude [21]. We thus speculate that our observed upregulation of MLCP and MLCK in LAL myocardium were associated with higher contractile strains in LAL LV. In an earlier study, Krejčí et al. conducted microarray analyses to investigate the biological processes associated with LAL. They found that secretion and extracellular region were significantly enriched among genes affected in left ventricles in LAL hearts at HH34. This corroborated with upregulation of MMPs, BMP2, TIPM3, FGF18, all types of Collagen and CAVIN1 in our results which are annotated to secretion and extracellular region biological processes.

The results of the present study are certainly subject to limitations. Firstly, limited experimental sample size used in this study because of substantial time needed for segmentations, motion tracking and FEM simulation for each sample. Second, ventricle trabeculation was not modeled during the tissue segmentation because high-frequency ultrasound did not have sufficient resolution to properly visualize trabeculation details. Thirdly, an assumption was made that the LV and RV had the same passive material properties in the FEM simulations. Fourthly, the biomechanics analysis was done at embryonic day 4.5, while gene analysis was done at embryonic day 6.5. Biomechanics analysis was done earlier because our hypothesis was that biomechanics stimuli preceded and caused disease gene expressions. However, due to the time gap, the biomechanics conditions in the heart might have changed nearer to 6.5 days. We were unable to image the embryonic heart close to 6.5 days as the embryo sank deeper into the egg, out of the field of view of our ultrasound probe. Further, in our cardiac active tension model in FE modeling, some parameters were kept constant between normal and LAL hearts due to a lack of data on them, such as initial sarcomere length, and time to peak tension, potentially leading to errors. Future work on how LAL might have changed these parameters can be conducted.

## Supplementary Information

Below is the link to the electronic supplementary material.

Supplementary file1 (MP4 887 KB)

Supplementary file2 (MP4 2841 KB)

Supplementary file3 (MP4 3947 KB)

Supplementary file4 (DOCX 453 kb)
