# Supplementary material for: Myocardial Biomechanics and the Consequent Differentially Expressed Genes of the Left Atrial Ligation Chick Embryonic Model of Hypoplastic Left Heart Syndrome
Source: Ann Biomed Eng. 2023 Apr 9;51(5):1063–78. doi: 10.1007/s10439-023-03187-0 (PMC10122626; doi:10.1007/s10439-023-03187-0)
Supplement: Supplementary file 4 — Supplementary file4 (DOCX 453 kb) [file 10439_2023_3187_MOESM4_ESM.docx]

**Supplementary Figures**


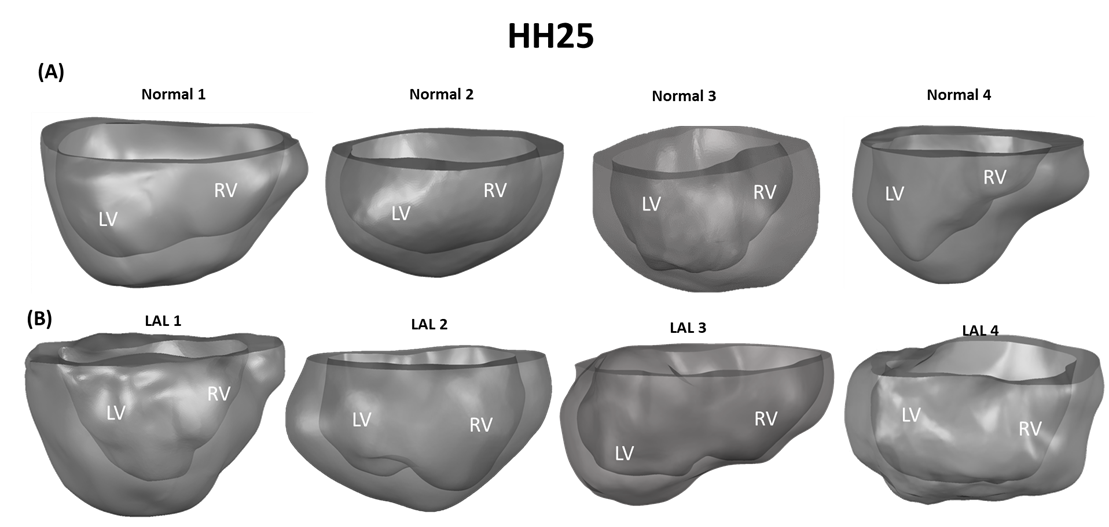


***Supplementary Figure 1:*** *End-systole 3D reconstructions of the tissue volumes of the chick embryonic ventricles at HH25 for* ***(A)*** *Normal hearts and* ***(B)*** *Left atrial ligated hearts.*


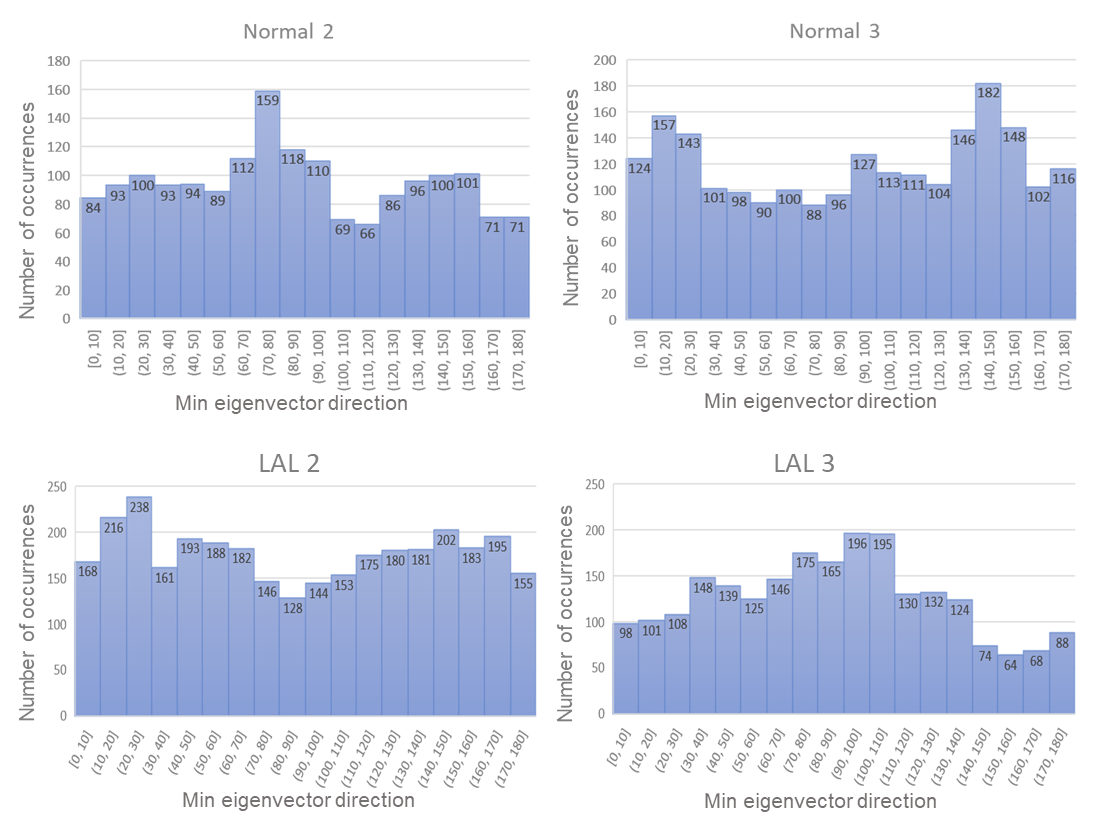


***Supplementary Figure 2:*** *Histogram of directions of minimum strain eigenvector, projected onto the circumferential-longitudinal plane, quantified in terms of deviation from the circumferential direction, for selected chick embryonic hearts. This was used as the indicator of the directions of active tension generation. This minimum eigenvector directions showed a uniform distribution with no preferred alignment, for both normal and LAL ventricles.*

| Sample No | Control | | LAL | |
| --- | --- | --- | --- | --- |
|  | m | b | m | b |
| 1 | 65 | -33 | 93 | -64 |
| 2 | 54 | -9 | 77 | -51 |
| 3 | 43 | 10 | 67 | -6 |
| 4 | 89 | -76 | 44 | 17 |

***Supplementary Table 1****: m and b constants in equation 6 that were used for all simulations*
